# A new perspective on protecting the blood-retinal barrier against injury in diabetic retinopathy: mitophagy

**DOI:** 10.3389/fendo.2025.1617797

**Published:** 2025-09-03

**Authors:** Mengtian Li, Liyang Yang, Haoyu Zhai, Liping Qiao, Zhaobo Wang, Xuedong An, Jia Wang

**Affiliations:** ^1^ Department of General Internal Medicine, Guang’anmen Hospital, China Academy of Chinese Medical Sciences, Beijing, China; ^2^ Beijing University of Chinese Medicine, Beijing, China; ^3^ Department of Oncology, Guang’anmen Hospital, China Academy of Chinese Medical Sciences, Beijing, China; ^4^ Jiangsu Provincial Hospital of Traditional Chinese Medicine Affiliated to Nanjing University of Chinese Medicine, Nanjing, China; ^5^ Department of Endocrinology, Guang’anmen Hospital, China Academy of Chinese Medical Sciences, Beijing, China

**Keywords:** diabetic retinopathy, blood-retinal barrier, mitochondrial dysfunction, mitophagy, inflammation, oxidative stress, therapeutic strategy

## Abstract

The blood-retinal barrier (BRB) comprises the inner blood-retinal barrier (iBRB) and the outer blood-retinal barrier (oBRB). The integrity of the BRB is essential to maintaining stability of the retinal microenvironment. Mitophagy plays a crucial role in maintaining organellar integrity by regulating mitochondrial quality and quantity. High glucose-induced mitophagy dysfunction contributes to diabetic retinopathy (DR) by damaging the BRB. This review presents mitophagy mechanisms under physiological conditions and examines changes across different cell types under DR-related pathological conditions that damage the BRB. It also summarizes drugs and targets that regulate mitophagy to stabilize the BRB and alleviate DR, offering new therapeutic insights.

## 1 Introduction

In 2015, approximately 415 million people worldwide were affected by diabetes, and this number is expected to reach 642 million by 2040 (1). DR is the most common microvascular complication of diabetes, with a prevalence of approximately 34.6% among patients with diabetes (2). Diabetic macular edema (DME), a complication of DR, affects 6.8% of patients with diabetes mellitus (DM) and is a leading cause of visual impairment and blindness. Clinically, DR is classified as proliferative (PDR) or non-proliferative (NPDR). NPDR is characterized by retinal capillary basement membrane thickening, increased retinal vascular permeability, and tissue ischemia, whereas PDR involves pathological neovascularization, potentially leading to vitreous hemorrhage or retinal detachment (3). The potential mechanisms causing these vascular issues include the breakdown of BRB. The integrity of the BRB helps keep the retinal microenvironment separate from the systemic circulation, reducing the impact of oxidative stress on this microenvironment, thereby maintaining its homeostasis under hyperglycemic conditions (4).

The BRB is composed of an inner layer and an outer layer. The iBRB mainly consists of a microvascular network that nourishes the retina’s inner layer. Its primary structure is vascular endothelial cells. These endothelial cells interact with neurons, pericytes, and glial cells to form the neurovascular unit (NVU) (5). The oBRB consists of a monolayer of retinal pigment epithelial (RPE) cells that interact with the fenestrated choroidal capillaries and Bruch’s membrane, whereas adjacent RPE cells are connected by tight junctions (6). The integrity of the BRB is essential for protecting the retina from harmful substances, clearing metabolic waste, regulating angiogenesis, and maintaining visual signal transmission.

Intravitreal anti-vascular endothelial growth factor (anti-VEGF) therapy is the established first-line treatment for center-involved DME (7). However, only 29% of patients show significant visual improvement after 2 years of treatment (8). Moreover, antibody-based therapies impose a significant economic burden on patients, making anti-VEGF therapy unlikely to be widely used for the routine treatment of NPDR. Given the limitations of current DR therapies, there is a need to explore new strategies targeting the core pathophysiological mechanisms. The disruption of the BRB, a critical event in the progression of DR, has molecular mechanisms that remain inadequately targeted by current therapies. Recent studies have shown that mitophagy dysfunction is a key factor in the destruction of the BRB during the progression of DR (9, 10). Focusing on the BRB, this review first analyzes the composition and molecular mechanisms underlying high glucose-induced BRB damage in DR, including mitochondrial dysfunction, inflammation, oxidative stress, and related pathways. Subsequently, it explores the regulatory mechanisms of mitophagy—a selective autophagy process that eliminates damaged mitochondria—and finally reviews potential drugs and targets aimed at ameliorating BRB damage through mitophagy modulation in DR, offering novel mechanistic insights and therapeutic strategies for targeting BRB lesions.

## 2 Composition and damage of the BRB

### 2.1 Composition of the BRB

The BRB consists of the iBRB and oBRB, which separate the systemic circulation from the retina (11). This unique structure not only helps transport nutrients and oxygen but also blocks large molecules, pathogens, and toxins from the blood. It also selectively regulates molecular flow between the systemic circulation and the retina, therefore maintaining homeostasis of the retinal microenvironment (12).

#### 2.1.1 Composition of the iBRB

Retinal endothelial cells (RECs), the principal components of the iBRB, constitute the retinal microvascular endothelium. These cells form a selective barrier via tight junctions between adjacent cells, regulating the transport of fluids and macromolecules between the blood and neural retina (13). The formation, maturation, and stability of retinal microvessels depend on the interactions between pericytes and endothelial cells. Specifically, RECs are interconnected by tight junction proteins and ensheathed by pericytes and glial cells (e.g., Müller cells and astrocytes) (14). Furthermore, endothelial cells within the retinal microvasculature rely on vascular endothelial (VE)-cadherin-mediated, calcium-dependent adherens junctions between the cells, which are essential for maintaining barrier integrity (15). Although the iBRB specifically reflects the specialized properties of RECs, the integrated functions of all NVU components, including neurons (ganglion, bipolar, horizontal, and amacrine cells), glial cells (astrocytes and Müller cells), immune cells (microglia), and vascular cells (RECs and pericytes), are essential for maintaining iBRB integrity and dynamically coordinating local blood flow to meet the metabolic demands of retinal neurons (16). Bidirectional signaling among these NVU cells constitutes a complex, dynamic network. The retinal vascular network regulates blood flow, angiogenesis, and permeability in response to the dynamic demands of retinal neurons. By providing oxygen and nutrients, recycling neurotransmitters, and clearing metabolic waste, the retinal vasculature plays a crucial role in fine-tuning vascular function to maintain retinal homeostasis (17). The endothelial cell population, specifically RECs, plays an important role in retinal diseases. Early intervention targeting RECs can help reduce the progression of retinal lesions and visual impairment.

#### 2.1.2 Composition of the oBRB

The oBRB consists of the choroid, Bruch’s membrane (BM), and RPE cells. The RPE is closely associated with the oBRB and directly constitutes the foundation of the neural retina (11). The RPE contributes to barrier function by forming tight junction complexes and providing metabolic support to the neural retina (18). This cell layer is essential for maintaining visual function by creating a selective barrier between the choroidal capillaries and the neural retina and regulating bidirectional solute transport, including nutrient delivery and metabolic waste clearance (11). Therefore, understanding RPE function is vital for developing effective prevention and treatment strategies for DR.

**Figure 1:**
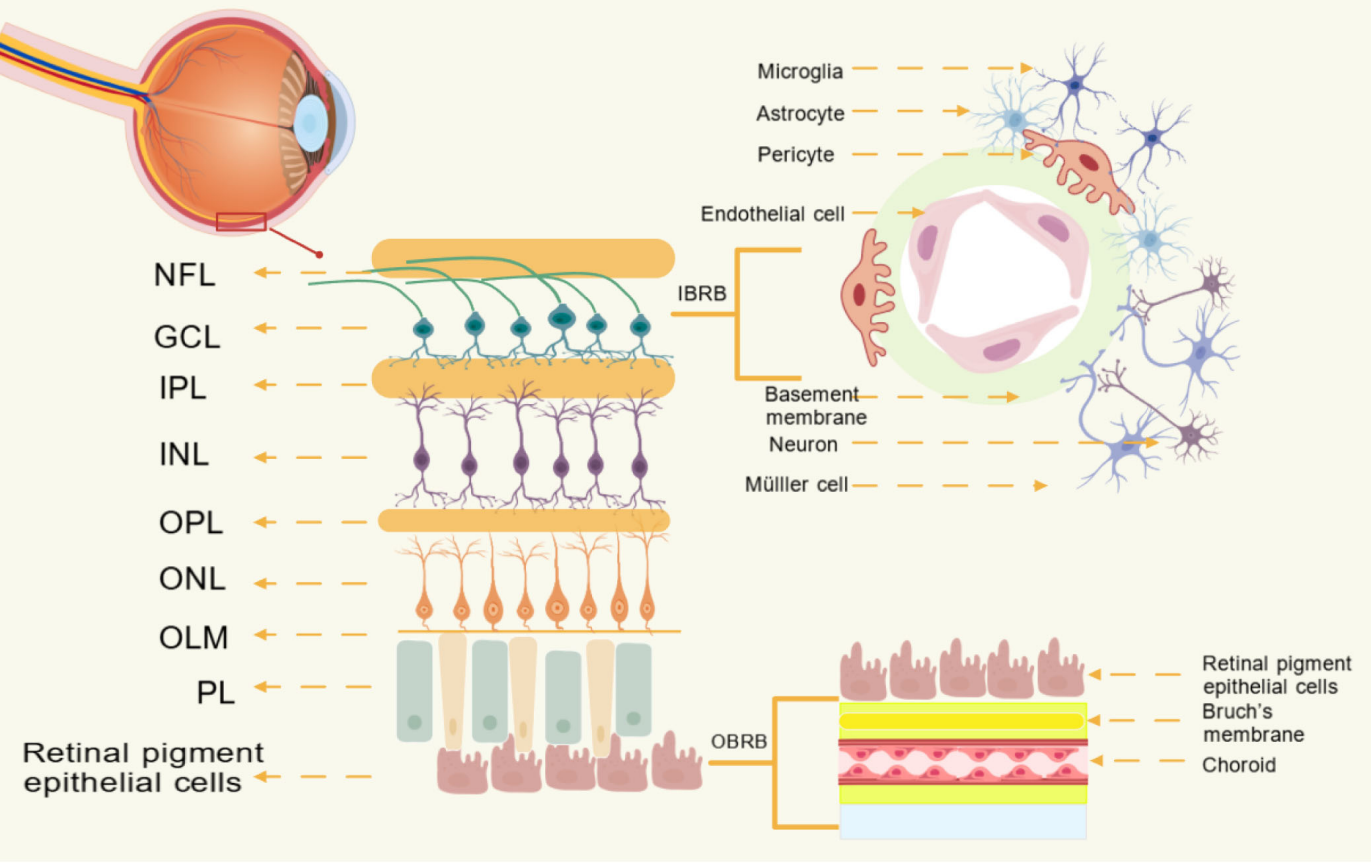
The image shows the structure of the blood-retinal barrier (BRB). The inner blood-retinal barrier (iBRB) is a functional barrier composed of retinal endothelial cells (RECs), which form retinal capillaries that traverse the inner layer of the retina. It is produced by vascular endothelial cells and features a double basement membrane, tight junctions between adjacent cells, and interactions with surrounding pericytes, microglia, and neurons. The retinal neurovascular unit (NVU), composed of vascular endothelial cells, pericytes, Müller glial cells, astrocytes, neurons, and microglial cells, contributes to the integrity of the iBRB. The outer blood-retinal barrier (oBRB) consists of the choroid, Bruch’s membrane (BM), and retinal pigment epithelium (RPE), with the RPE being most relevant to the oBRB, directly forming the foundation of the neuroretina. PL, Photoreceptor Layer; OLM, Outer Limiting Membrane; ONL, Outer Nuclear Layer; OPL, Outer Plexiform Layer; INL, Inner Nuclear Layer; IPL, Inner Plexiform Layer; GCL, Ganglion Cell Layer; NFL, Nerve Fiber Layer.

However, in the diabetic state, persistent pathological factors, including chronic hyperglycemia, severely disrupt the integrity and precise regulatory functions of both the iBRB and oBRB. This BRB dysfunction is one of the core pathophysiological bases for the onset and progression of DR (19) **Figure 1**.

### 2.2 Damage to the BRB in DR

The damage to the BRB in DR results from the multimechanistic synergy of four core pathways: inflammatory activation, oxidative stress, dysregulation of vascular growth factors, and mitochondrial homeostatic imbalance. These mechanisms are intertwined and mutually reinforcing, collectively driving disease progression.

#### 2.2.1 Inflammation

DR is a chronic, low-grade inflammatory disease involving various inflammatory mediators and adhesion molecules, with chronic hyperglycemia serving as the main trigger (20). Studies have shown that levels of interleukin-1β (IL-1β) and tumor necrosis factor-α (TNF-α) are elevated in the serum and vitreous of DR patients (21). IL-1β and TNF-α act as inducers of adhesion molecule expression by binding to their respective receptors (IL-1 receptor and TNF receptor), thereby promoting the activation of nuclear factor kappa-B (NF-κB). This activation leads to increased expression of IL-6 and IL-8, and it also triggers caspase-1 activation (22). Furthermore, IL-1β activates NF-κB, which enhances the adhesion of retinal capillary cells to the endothelium and induces their apoptosis. These events, therefore, increase vascular permeability (22, 23). TNF-α promotes leukocyte adhesion to RECs, directly impairing BRB integrity (24, 25). These pro-inflammatory cytokines stimulate RECs to upregulate intercellular adhesion molecules, thereby facilitating leukocyte recruitment and capillary adhesion. Adherent leukocytes obstruct capillaries and disrupt tight endothelial junctions. Consequently, BRB dysfunction manifests as acellular capillary formation, vascular leakage, and DME (25). Furthermore, hyperglycemia (HG) activates the NLRP3 (NOD-, LRR-, and pyrin domain-containing protein 3) inflammasome, contributing to inflammation. Activation of the NLRP3 inflammasome mediates cytokine secretion, which downregulates tight junction protein expression and exacerbates BRB damage (26).

#### 2.2.2 Oxidative stress

In the hyperglycemic state of diabetes, chronic hyperglycemia causes oxidative stress through four main pathways, generating reactive oxygen species (ROS) and exacerbating damage to the BRB. One such pathway is the activation of the polyol pathway of glucose metabolism (27), where aldose reductase (AR) converts glucose into sorbitol, which is then oxidized by sorbitol dehydrogenase into fructose. During this process, the cofactor nicotinamide adenine dinucleotide (NAD^+^) is reduced to NADH (28, 29). This leads to an abnormal increase in the NADH/NAD+ ratio, with excess NADH acting as a substrate for NADPH oxidase, thereby promoting ROS generation within retinal cells (30). Products generated by fructose phosphorylation and degradation can serve as precursors for the formation of advanced glycation end products (AGEs) (31). HG-induced accumulation of diacylglycerol activates the protein kinase C (PKC) pathway and promotes the expression of VEGF in retinal tissues (32). The upregulation of VEGF contributes to endothelial dysfunction, increased vascular permeability, and pathological neovascularization in DR (33). Additionally, PKC enhances the activity of NADPH oxidase, thereby promoting ROS production in various vascular cells (34). Prolonged hyperglycemia significantly increases the non-enzymatic glycation of proteins and lipids, ultimately leading to AGEs accumulation (35). The interaction of AGEs with their receptor RAGE promotes NF-κB activation, triggering retinal pericyte apoptosis and upregulating VEGF, which increases vascular endothelial permeability (36).Additionally, this AGEs-RAGE axis stimulates Müller cells to release VEGF and MCP-1 (monocyte chemoattractant protein-1), further disrupting the BRB and inducing inflammatory infiltration (37). Furthermore, AGEs-RAGE interaction activates NADPH oxidase, enhancing intracellular ROS generation (38, 39). Elevated ROS levels, in turn, contribute to AGEs formation, further amplifying AGEs-mediated damage (40). AGEs stimulation can also lead to the upregulation of proteases, which cleave VE-cadherin on RECs, resulting in BRB breakdown and increased vascular permeability (41). After activation of the hexosamine pathway, the resulting high concentrations of glucosamine accumulate, stimulating excessive ROS generation in mitochondria and damaging mitochondrial respiratory function. This process further exacerbates oxidative stress, increases vascular permeability, and promotes angiogenesis (42, 43).

ROS are continuously produced in all cells to support normal cellular functions. However, under hyperglycemic conditions, the activation of the four pathways mentioned above stimulates excessive production of mitochondrial ROS, further exacerbating oxidative stress. First, elevated ROS levels activate the NLRP3 inflammasome in RECs, as induced by AGEs in diabetic rats. This activation leads to the caspase-1-dependent release of the pro-inflammatory cytokine IL-1β (44). These inflammatory cytokines damage RECs, impairing their ability to maintain BRB integrity. Second, heightened oxidative stress potently activates NF-κB, which regulates the transcription of numerous genes. The expression of zonula occludens-1 (ZO-1), a critical tight junction protein, is suppressed by NF-κB, thereby disrupting the normal structure of the BRB (45). Furthermore, oxidative stress, which is exacerbated by excess ROS, contributes to mitochondrial dysfunction. Notably, mitochondrial DNA (mtDNA) is particularly vulnerable to extensive and persistent damage induced by oxidative stress (46). Damaged mtDNA impairs transcription and protein synthesis, resulting in further production of ROS.

#### 2.2.3 Elevated vascular endothelial growth factor

Elevated VEGF levels are positively correlated with DR severity, particularly in PDR. Studies have demonstrated significantly higher serum VEGF concentrations in patients with PDR than in patients with NPDR and healthy controls (47). Similarly, the aqueous humor VEGF levels in patients with PDR substantially exceed those in non-DR individuals (48). Firstly, in a vitro co-culture model of the BRB based on primary RECs, pericytes and astrocytes, VEGF treatment disrupts the junctional assembly of tight junction proteins (occludin, claudin-5, ZO-1), adherens junction protein (VE-cadherin), directly compromising BRB structural integrity (49).Elevated VEGF levels also induce the expression of urokinase plasminogen activator receptor (uPAR), leading to activation of matrix metalloproteinase-9 (MMP-9), which degrades the extracellular matrix and further disrupts junctional complexes (50). Secondly, VEGF significantly increases the expression of plasmalemma vesicle-associated protein (PLVAP), promoting endothelial cell vesicular transport activity. This facilitates plasma protein leakage into retinal tissue via the transcellular route (49, 51). Furthermore, VEGF induces RECs proliferation, promoting retinal neovascularization, which ultimately progresses to PDR (52).

#### 2.2.4 Imbalance in mitochondrial dynamics and mitophagy

Mitochondria are key organelles responsible for energy production and serve as central hubs for nutrient metabolism. They play vital roles in metabolism, signal transduction and are essential for cellular processes including growth, differentiation, aging, and death. Within the mitochondrial inner membrane, NADH and flavin adenine dinucleotide (FADH_2_) act as electron donors, transferring electrons to the electron transport chain (ETC). During electron transfer, protons (H^+^) are pumped into the intermembrane space, generating an electrochemical gradient. This gradient drives ATP synthesis by ATP synthase, producing chemical energy stored in ATP molecules that fuel cellular processes. Maintaining mitochondrial homeostasis critically depends on the precise regulation of dynamic processes, namely mitochondrial fusion, fission, and mitophagy. Fusion preserves mitochondrial network integrity and facilitates the complementation of damaged mtDNA (53, 54). Fission segregates damaged or dysfunctional mitochondria, enabling their subsequent incorporation into autophagosomes (55). Furthermore, mitochondria that exhibit a reduced membrane potential following fission are selectively engulfed by autophagosomes and degraded via mitophagy (56), thereby ensuring mitochondrial quality control and homeostasis (57). Mitophagy specifically targets and degrades damaged mitochondria through a lysosome-dependent pathway (58, 59). This extensive clearance of damaged mitochondria prevents ROS generation and accumulation, halts the propagation of mitochondrial damage, and confers cytoprotection (60). Under conditions of cellular damage or stress, mitophagy reduces the burden of mitochondria harboring pro-death signals through targeted elimination, thereby delaying the onset of apoptosis. Furthermore, mitophagy regulates apoptotic cascades via cytochrome c release, which activates caspase-family cysteine proteases to initiate programmed cell death (61). Mitophagy also modulates inflammatory responses and oxidative stress. Inflammatory diseases are primarily mediated by inflammasome activation such as NLRP3 (62, 63). By controlling the release of mitochondrial-derived damage-associated molecular patterns (mtDNA and ROS), mitophagy regulates inflammasome activation. It also exhibits cell-intrinsic anti-inflammatory mechanisms by suppressing excessive production of IL-1β and IL-18. Therefore, mitochondrial fusion, fission, and mitophagy collectively maintain efficient ATP generation while constraining ROS accumulation (64).

Mitochondrial number, size, structure, and physiological functions are typically altered under pathological conditions, including ischemia, hypoxia, nutrient deficiency/imbalance, endotoxin exposure, and calcium overload (32). In DR, imbalanced mitochondrial dynamics manifest as enhanced fission and suppressed fusion, promoting the intracellular accumulation of damaged or dysfunctional mitochondria (65). Concurrently, hyperglycemia may initially elevate mitophagic flux; however, when mitophagic activity exceeds the lysosomal degradative capacity, lysosomal enlargement occurs, accompanied by diminished enzyme activity, resulting in impaired clearance of damaged mitochondria (66). In addition, other studies have indicated that sustained high-glucose conditions ultimately suppress mitophagy and cellular proliferation (67). This mitophagy inhibition amplifies ROS overproduction (68), induces mtDNA mutations, disrupts cellular architecture, impairs metabolic homeostasis, and potentiates apoptosis (69, 70). Oxidative stress and calcium overload can trigger mitochondrial permeability transition (mPT) through the mitochondrial permeability transition pore (mPTP), facilitating the efflux of cytochrome c and other mitochondrial components. This process initiates apoptotic cascades and inflammatory responses (71). Persistent mPTP opening depletes NADH reserves, damages respiratory chain complex I, and further exacerbates oxidative injury (72, 73). Notably, chronic hyperglycemia may drive TXNIP-mediated sustained ROS accumulation and progressive ATP depletion, ultimately causing excessive mitochondrial elimination and worsening cellular dysfunction (74).

In the aforementioned mitochondrial dynamics disorders and related pathological cascades, mitophagy is the core mechanism for clearing damaged mitochondria, and its function directly affects cellular homeostasis. Under normal circumstances, it maintains mitochondrial population homeostasis by degrading abnormal mitochondria; however, in the hyperglycemic environment of DR, mitophagy may become functionally imbalanced due to flux overload, pathway inhibition, or excessive activation, which not only fails to prevent the accumulation of damaged mitochondria but also amplifies oxidative stress and exacerbates BRB damage. Therefore, clarifying the molecular mechanisms and pathological changes of mitophagy is crucial for understanding the progression of DR and developing targeted therapeutic strategies.

## 3 Mechanisms of mitophagy

Under mitochondrial damage conditions, mitophagy becomes an essential cellular quality control mechanism and includes two main categories: the ubiquitin (Ub)-dependent and Ub-independent pathways.

Ub-dependent pathway: This primarily involves the PINK1/Parkin pathway (75). Parkin is a cytosolic E3 ubiquitin ligase that links Ub molecules to substrate proteins. The identification of cytosolic ubiquitinated substrates of Parkin has advanced, and studies have demonstrated that Parkin plays a protective role in stabilizing mitochondrial function and morphology (76). Early studies regarded PINK1 as a protein homologous to PTEN, which is believed to be associated with tumors and Parkinson’s disease (77). PINK1 is widely recognized as a highly conserved mitochondrial protein involved in various cellular and physiological processes, primarily regulating mitochondrial function (78). PINK1 is autophosphorylated in the outer mitochondrial membrane (OMM), which is crucial for its activation and accumulation in mitochondria (79). In healthy mitochondria, PINK1 is degraded by other proteins after entering the inner membrane, resulting in low detectable levels (80). However, when mitochondria are damaged, changes in membrane structure or potential (ΔΨm) inhibit PINK1 degradation, blocking its entry into the inner membrane, leading to increased accumulation of PINK1 on the OMM (81). The accumulation of PINK1 in the OMM promotes the translocation of Parkin to the mitochondria, thereby inducing mitophagy in dysfunctional mitochondria (82). Additionally, PINK1 phosphorylates ubiquitin at the Ser65 site to activate Parkin’s ubiquitin ligase activity, recruiting more autophagy receptors. The recruitment of receptors such as NDP52 and OPTN can facilitate mitophagy (83). Subsequently, light chain 3 (LC3) is recruited to damaged mitochondria through DFCP1, allowing OPTN to mediate the initiation of autophagosomes through LC3 interaction regions (LIR) (84, 85). Ubiquitinated mitochondria can be recognized by the ubiquitin-binding protein P62. Subsequently, P62 binds to microtubule-associated LC3, allowing damaged mitochondria to be engulfed by autophagosomes and ultimately degraded by lysosomes. This process promotes mitophagy (86).

**Figure 2:**
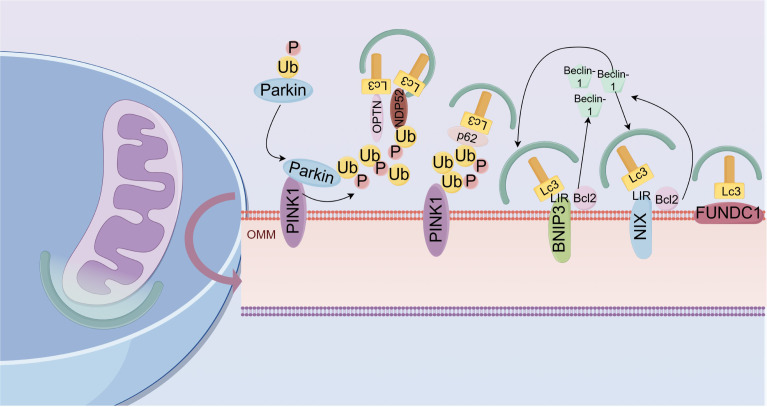
Schematic diagram of the mechanisms of mitophagy: Mitophagy is regulated through both ubiquitin (Ub)-dependent and -independent pathways. The Ub-dependent pathway is dominated by the PINK1/Parkin pathway: When mitochondria are damaged, PINK1 accumulates on the outer mitochondrial membrane (OMM) and phosphorylates Ub, activating Parkin’s ubiquitin ligase activity, promoting the ubiquitination of OMM proteins; the ubiquitination signal recruits autophagy receptors (NDP52, OPTN), connecting to autophagosomes through LC3 interaction regions (LIR), which are ultimately degraded by lysosomes. The Ub-independent pathway includes BNIP3/Nix and FUNDC1 pathways: BNIP3 and NIX interact with Atg8 on autophagosomes through their LIR, thereby inducing mitophagy. NIX and BNIP3 can also interact with Bcl-2, increasing the cytoplasmic levels of free Beclin-1 and promoting the activation of Atg5, ultimately initiating mitophagy. Furthermore, FUNDC1 is another protein located on the OMM that can induce mitophagy by binding to LC3 under hypoxic conditions.

Unlike the PINK1/Parkin pathway, the BNIP3/Nix pathway mediates mitophagy independently of ubiquitination.Bcl-2 and adenovirus E1B 19-kDa interacting protein 3 (BNIP3) and BNIP3-like protein (BNIP3L, also known as Nix) are homologous members of the Bcl-2 family of proteins (87). They are located on the OMM and were initially classified as apoptotic proteins, functioning as engulfment receptors under cellular developmental or pathological conditions.BNIP3 is present at low abundance in cells but is upregulated under hypoxia. Hypoxia-inducible factor-1α (HIF-1α), an important transcription factor affecting mitophagy, can upregulate BNIP3 and NIX under hypoxic conditions, thereby clearing excessive mitochondria and maintaining cell viability (88). BNIP3 acts as a hypoxia-induced mitochondrial connector that directly targets mitochondrial structures (89). The NIX protein directly binds LC3 through its BH3 domain and induces mitophagy (90, 91). Both BNIP3 and NIX contain LC3 interaction regions (LIRs) at their N-termini, which bind to the homolog of autophagy-related gene 8 and induce mitophagy (92). NIX and BNIP3 also interact with Bcl-2, thereby increasing the cytoplasmic levels of free Beclin-1 and promoting the activation of autophagy-related gene 5, ultimately initiating mitophagy (92). Fun14 domain-containing protein 1 (FUNDC1) is another protein located on the OMM that, similar to BNIP3/NIX, can induce mitophagy by binding to LC3 through its LIR under hypoxic conditions (93) (**Figure 2**).

Mitophagy plays a central role in clearing damaged mitochondria through both ubiquitin-dependent (PINK1/Parkin pathway) and -independent (BNIP3/Nix and FUNDC1 pathways) mechanisms, maintaining cellular homeostasis and regulating apoptosis and inflammation. In DR, metabolic disorders induced by hyperglycemia directly interfere with the normal functioning of these mechanisms in cells, leading to mitophagy dysfunction and subsequent BRB damage. This article specifically elaborates on the pathological changes in mitophagy in the core constituent cells of the BRB (such as RPE cells, RECs, pericytes, and Müller cells) under DR conditions and their impact on barrier integrity.

## 4 Mitophagy dysfunction in BRB constituent cells: a mechanism underlying barrier injury in DR

Increasing evidence suggests that mitochondrial dysfunction plays a key role in the pathogenesis of DR. Specifically, excessive mtROS production, mtDNA damage, ETC damage, and inefficient mtDNA repair mechanisms caused by mitochondrial dynamic imbalance all contribute to DR (94). In particular, mitophagy, as the core mechanism for clearing damaged mitochondria, is crucial for maintaining the homeostasis of BRB constituent cells. Under high-glucose conditions, mitophagy in cells may undergo bidirectional changes. Excessive mitophagy can remove essential organelles and proteins, leading to a loss of compensatory capacity and ultimately resulting in apoptosis (95). Conversely, a decrease in mitophagy can lead to the accumulation of damaged mitochondria, resulting in BRB degradation (10).

### 4.1 BRB damage caused by excessive activation of mitophagy

RECs are the main components of the iBRB, and their dysfunction plays an important role in the pathogenesis of DR. Hyperglycemia disrupts mitochondrial homeostasis in RECs, leading to their dysfunction. The destruction of connections between adjacent RECs and the apoptosis of RECs are the primary drivers of the acellular capillary formation and the subsequent destruction of the iBRB during NPDR (96). Studies on HG intervention in rat RECs revealed that HG upregulates dynamin-related protein 1 (Drp1). Following its activation, Drp1 induces excessive mitochondrial fission, generating numerous fragmented mitochondria. These fragmented mitochondria are prone to damage, releasing ROS and triggering mitophagy, which ultimately leads to an elevated apoptosis rate in RECs (97). This directly disrupts the BRB, increasing vascular permeability. Additionally, oxidative stress further damages intercellular tight junctions, exacerbating vascular leakage of the BRB. Similarly, another study showed that mitophagy increased in retinal Müller cells cultured under HG conditions. The possible mechanism is that TXNIP induces mitochondrial oxidative stress and dysfunction, promoting Drp1-mediated fission and Parkin-mediated ubiquitination. Simultaneously, TXNIP inhibits ATG4B, enhancing autophagosome formation and inducing excessive mitophagy (98),ultimately leading to Müller cell dysfunction. As critical BRB supporting cells, their dysfunction impairs the metabolic and trophic support to RECs and retinal neurons, further compromising BRB integrity.

### 4.2 BRB damage caused by inhibition of mitophagy

However, many scholars have observed the inhibitory effects of mitophagy pathways in DR. HG induces mitochondrial fission by activating PKCδ/Drp1 in RECs, resulting in damaged mitochondria. Additionally, HG phosphorylates Drp1 via PKCδ, triggering the dissociation of HK-II from the outer mitochondrial membrane and blocking HK-II-mediated activation of the PINK1/Parkin pathway. As a result, HK-II-mediated PINK1/Parkin mitophagy is inhibited, leading to RECs apoptosis and subsequent damage to the barrier (99). Moreover, in microglia treated with HG (BV2 cell model), HG upregulated Poldip2 expression, promoting the ubiquitination degradation of Pink1 and inhibiting mitophagy, leading to the accumulation of dysfunctional mitochondria that cannot be cleared (100). Damaged mitochondria accumulated in microglia trigger oxidative stress and endoplasmic reticulum stress. These stress responses activate microglia to polarize toward the pro-inflammatory M1 type, releasing large amounts of inflammatory cytokines including IL-6 and TNF-α. These cytokines damage the tight junctions of the BRB, increasing vascular permeability and leading to pathological changes such as BRB dysfunction, vascular leakage, and neovascularization. Research using AGEs to simulate the diabetic microenvironment in rat Müller cells observed similar trends in mitophagy changes. Specifically, levels of TOM20, LC3II/LC3I, PINK1, and Parkin proteins were significantly downregulated, whereas P62 levels were elevated. These changes indicate that AGEs inhibit the mitophagy function of Müller cells (101). Excessive ROS production induces apoptosis and glial activation in Müller cells. As key supporting cells of the BRB, Müller cell glial activation disrupts the physical connections and functional synergy of retinal vascular endothelial cells, weakening the structural stability of the BRB. Increased oxidative stress and the release of inflammatory cytokines enhance retinal vascular leakage, ultimately leading to BRB functional collapse.

Treatment with HG (50 mM) reduces PINK1/Parkin signaling in the RPE by elevating ROS levels, leading to increased apoptosis of the RPE, reduced proliferation, and exacerbated oxidative stress, ultimately compromising the integrity of the BRB (67). Sirtuin 3 (SIRT3) is a mitochondrial NAD+-dependent deacetylase that plays a key role in mitochondrial metabolic regulation (102). Studies have found that HG leads to decreased SIRT3 expression, inhibiting the AMPK/mTOR/ULK1 pathway and resulting in reduced mitophagy (103). Inhibition of mitophagy triggers ROS accumulation and apoptosis in RPE, potentially compromising the integrity of the BRB. HG causes the accumulation of Telomeric Repeat-Binding Factor 1 (TRF1)-interacting protein 2 (TIN2) in mitochondria in RPE cells. The accumulation of TIN2 reduces the expression of mitophagy-related proteins Microtubule-associated protein 1 light chain 3 beta(LC3B), PINK1, and Parkin, thereby inhibiting mitophagy. The inhibition of mitophagy disrupts the tight junctions of RPE cells, leading to structural damage of the BRB (104). The accumulation of AGEs caused by hyperglycemia is a major factor in the development of DR, and Methylglyoxal(MGO) is a precursor of AGEs (105). MGO levels in diabetic patients are higher than those in healthy controls (106), potentially leading to impaired retinal blood flow regulation in patients with DR (107)MGO suppresses protein and gene expression of mitochondrial fusion protein-1, peroxisome proliferator-activated receptor gamma coactivator 1-alpha, and mitochondrial transcription factor A, thereby reducing mitochondrial biogenesis and fusion. Concurrently, MGO inhibits AMP-activated protein kinase (AMPK) activity, decreases LC3-II accumulation, and impairs mitophagy in RPE cells, ultimately leading to RPE cells death (108).

**Figure 3:**
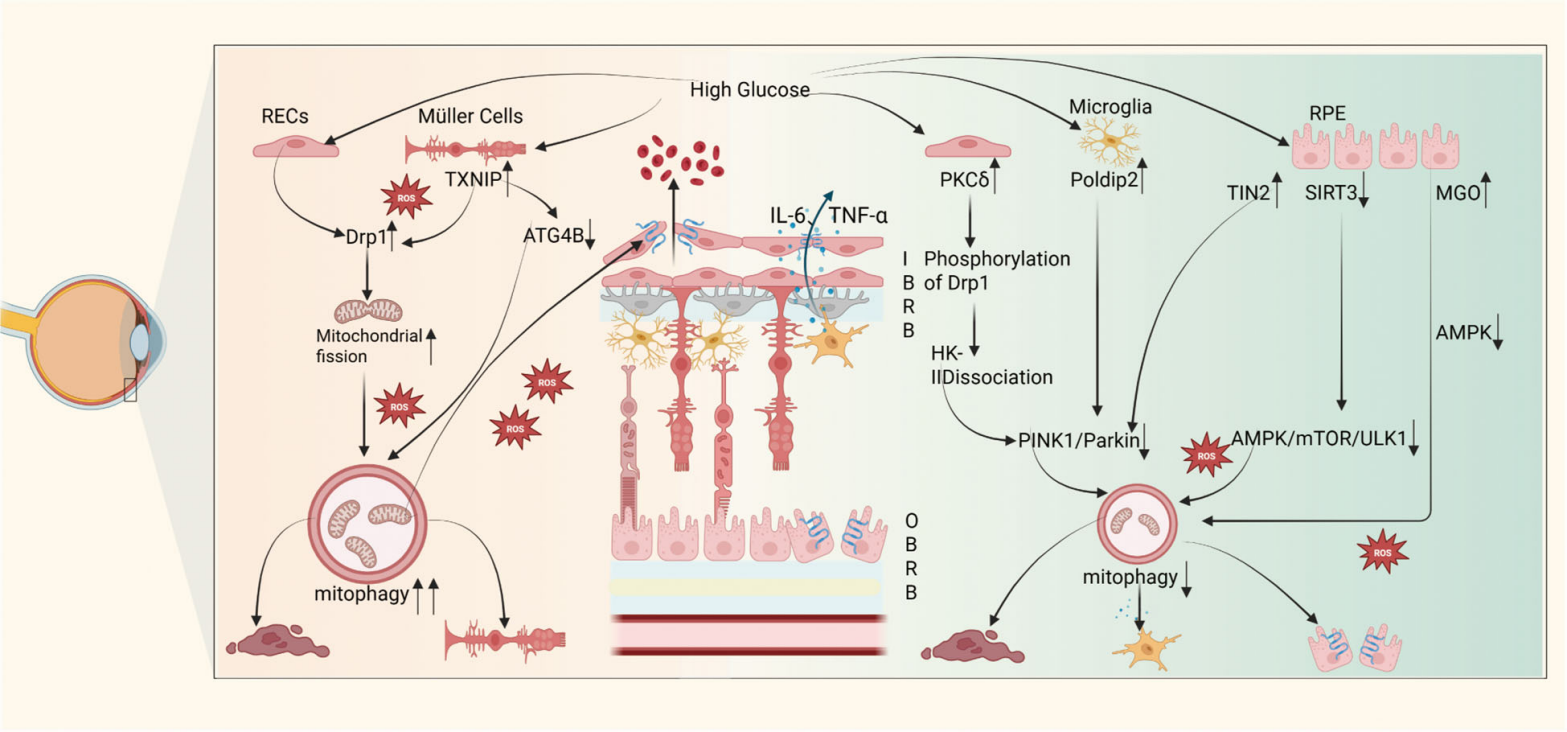
HG induces impairment of mitophagy in BRB constituent cells, contributing to BRB disruption in DR. The left panel depicts HG-promoted mitochondrial fission, resulting in oxidative stress and excessive mitophagy activation, ultimately culminating in RECs apoptosis and Müller cell death. In RECs, HG phosphorylates Drp1 via PKCδ, triggering HK-II dissociation from mitochondria and blockade of the PINK1/Parkin pathway, thereby suppressing mitophagy, inducing damaged mitochondrial accumulation, and promoting REC apoptosis. In microglia, HG promotes pro-inflammatory M1 polarization, inducing substantial secretion of inflammatory cytokines (e.g., IL-6, TNF-α), thereby compromising BRB tight junctions and increasing vascular permeability. In RPE cells, HG inhibits mitophagy through suppression of both PINK1/Parkin and AMPK/mTOR/ULK1 pathways, driving RPE apoptosis and tight junction disruption. Collectively, these HG-driven impairments compromise BRB integrity and increase vascular permeability.

The inconsistencies in cellular mitophagy changes in DR may be owing to the level of mitophagy depending on the degree of hyperglycemia. Zhang et al. observed that in cultured retinal pigment epithelial cells, a slight increase in glucose concentration (15 mM) induced upregulation of mitophagy, whereas a significant increase (50 mM) inhibited mitophagy, leading to apoptosis (67). The corresponding mechanism may be that mild hyperglycemia induces a stress response, prompting cells to clear damaged mitochondria through mitophagy. Conversely, severe or persistent hyperglycemia causes cellular damage, leading to mitophagy dysfunction. Additionally, the duration of diabetes appears to play a crucial role, as studies on human retinas, mice, and primary Müller cells have demonstrated that prolonged diabetes progressively suppresses Pink1-dependent mitophagy, causing the accumulation of damaged mitochondria and eventually leading to BRB breakdown (10). Furthermore, as the duration of diabetes increases, aging may also affect mitophagy, as late-stage DR retinas show increased activity of senescence-associated β-galactosidase (SA-β-Gal). *In vitro* studies have shown that in continuously aged Müller cells, high-glucose levels, hyperosmolarity, or starvation fail to activate autophagy (10) **Figure 3**.

## 5 Therapeutic strategies targeting mitophagy dysfunction in BRB constituent cells: protecting against BRB injury in DR

Intensified control of blood pressure and blood glucose levels reduces the incidence or slows the progression of microvascular complications in diabetic patients (e.g., DR), thereby preserving visual stability (109). Furthermore, a meta-analysis demonstrated that maintaining adequate glycemic control may mitigate the risk of progression to PDR and other vision-threatening complications, irrespective of baseline DR severity (110). Notably, DME, which is characterized by BRB disruption during DR progression, represents the leading cause of vision loss in patients with DR (111). Currently, several new methods for treating DME have been introduced, with anti-VEGF drugs being the first-line treatment for center-involved DME. Although anti-VEGF drugs maintain the integrity of the BRB by inhibiting VEGF-A and/or placental growth factor, some patients show a poor response to this treatment in clinical practice. Studies have shown that 31.6% to 65.6% of DME patients still exhibit persistent edema symptoms after receiving at least four regular intravitreal injections within 24 weeks (112). Furthermore, repeated injections are often required for many patients, which may pose economic burdens. Real-world evidence suggests that the mean annual injection frequency is approximately 3.1 sessions per patient, with about 68.6% of patients receiving ≤3 injections, generally below the dosing schedules used in clinical trials (typically 9–12 sessions) (113). For individuals with PDR, laser photocoagulation continues to be widely utilized as a therapeutic modality (114). It should be noted that laser treatment can sometimes be associated with discomfort, and extensive applications might potentially affect peripheral visual fields, particularly when involving central macular areas (115). Given the limitations of the aforementioned treatment methods, identifying new approaches to treat DR is particularly important. The previous discussion highlighted the key role of the BRB in retinal stability and the damage caused by mitophagy dysregulation in DR. The following section introduces existing research on maintaining cellular homeostasis by regulating mitophagy, aiming to provide new strategies for DR treatment from the perspective of preserving BRB integrity (**Table 1**).

**Table 1:** Protective effects of targeting mitophagy in BRB constituent cells on the BRB in DR.

Drug/targets	Models (cells or animals)/intervention measures	Influence pathways	Effects on mitophagy	Findings	Reference
Inhibition of mitophagy					
Melatonin	ARPE-19 (pretreated with HG+ deferoxamine mesylate, followed by melatonin treatment for 48 hours)	HIF-1α, HIF-1β, VEGF ↓;DRP1↓;PINK, BNip3, NIX↓;PGC-1α, NRF2↑	Inhibition of overactivated mitophagy	Melatonin reduces RPE cells apoptosis, alleviates BRB leakage, and enhances BRB integrity.	(118)
TXNIP	rMC1/STZ-induced SD rats(TXNIP knockout cell line/intravitreal injection of TXNIP siRNA)	ROS↓;DRP1↓;LC3BII puncta , Parkin↓; co - localization of COXIV and LAMP2A↓, OPTN, p62↓;GFAP↓	Inhibition of overactivated mitophagy	Knocking out TXNIP reduces apoptosis and gliosis in Müller cells, thereby BRB integrity and mitigating structural damage to the BRB in DR.	(98)
TXNIP	ARPE-19 (TXNIP knockout strain shTXNIP3 + 4)	ROS↓;Restore Trx1/Trx2 function, inhibit mitochondrial fission and TBK1-mediated phosphorylation of autophagy adaptors	Reduced mitophagic flux impairs autophagic degradation of damaged mitochondria	Knocking out TXNIP reduces RPE cells apoptosis, restores proliferative capacity, maintains RPE layer integrity, and protects the barrier function of BRB.	(66)
WIF1	ARPE-19/STZ-induced diabetic C57BL/6J mice (treated with WIF1/intravitreal injection of WIF1)	AMPK/mTOR, PINK1/Parkin↓;LC3-II/LC3-I , p62↓;ROS, MDA↓;SOD, GPX↑	Inhibition of overactivated mitophagy	WIF1 restores the function of RPE cells, reduces the formation of RECs tube, protects the integrity of BRB, and thickens all retinal layers	(120)
Promotion of mitophagy					
MSC-derived small extracellular vesicles (containing miR-125a-5p)	Rat Müller cells/STZ-induced SD rats (co-cultured with MSC-sEVs/intravitreally injected with MSC-sEVs)	PINK1/Parkin ↑;LC3II/LC3I , TOM20↑;P62 ↓;GFAP↓;occludin↑	Promotion of mitophagy	MSC-sEVs-miR-125a-5p reduce Müller cells apoptosis, restore their proliferative capacity, decrease retinal vascular leakage, and improve BRB integrity.	(101)
Leflunomide	HRECs(leflunomide added when normal blood glucose is restored after HG exposure)	Mfn2 ↑;ROS↓;LC3II/LC3I ↑	Promotion of mitophagy	Leflunomide reduces RECs apoptosis, improves their proliferative capacity, and protects BRB integrity.	(65)
Drp1	RMECs/STZ-induced SD rats (pretreated with Mdivi-1/intravitreally injected with Mdivi-1)	HK-II↑; PINK1/Parkin↑;LC3B-II↑, p62↓	Promotion of mitophagy	Inhibiting Drp1 can reduce RMECs apoptosis and retinal vascular leakage, decrease the number of acellular capillaries, restore retinal thickness, and protect BRB integrity.	(99)
TIN2	ARPE-19/STZ-induced C57BL/6J diabetic mice (transfected with sh-TIN2/intravitreally injected with aav-shTIN2)	mTOR↓;PINK1/Parkin↑;LC3B-II↑, p62↓;SA-β-gal positive cells↓;ZO-1↑	Promotion of mitophagy	Knocking out TIN2 inhibits RPE cells senescence; alleviates oxidative stress, protects BRB integrity, and increases retinal thickness.	(104)
Sirt3	ARPE-19(Transfection withLV-Sirt3 )	AMPK ↑, mTOR↓;ULK1↑, LC3B-II/LC3B-I ↑;ROS↓	Promotion of mitophagy	Sirt3 overexpression reduces RPE cells ROS and apoptosis, protects RPE cells integrity, and maintains BRB barrier function.	(103)
Poldip2	BV2 cells/STZ-induced diabetic SD rats (Transfection with Poldip2-siRNA/intravitreally injected with AAV9-Poldip2-shRNA)	AMPK/ULK1/Pink1/Parkin↑;IL-6, TNF-α ↓;VEGFR ↓;LC3B-II/LC3B-I ↑;p62 ↓	Promotion of mitophagy	Inhibition of Poldip2 increases microglial M2 polarization, reduces cytokines factors, decreases retinal vascular leakage, inhibits neovascularization, and protects BRB integrity.	(100)
TGR5	RMECs/STZ-induced SD rats (pretreated with INT-777/intravitreally injected with INT-777)	PKCδ/Drp1 ↓;HK2/PINK1/Parkin↑;LC3B-II/LC3B-I ↑;p62 ↓	Promotion of mitophagy	Activating TGR5 reduces retinal vascular leakage, RECs apoptosis and acellular capillaries, restores retinal thickness, and maintains BRB integrity.	(121)
VDAC1	HRCECs (Transfection with VDAC1-overexpressing adenovirus)	PINK1/Parkin↑; mtROS ↓; NLRP3 ↓	Promotion of mitophagy	VDAC1 overexpression reduces HRCECs proliferation, migration and tube formation, promotes apoptosis, and protects BRB integrity.	(122)
NGR1	rMC-1 cells/db/db mice (NGR1 pretreatment/oral administration of 30 mg/kg NGR1 for 12 weeks)	PINK1/Parkin↑; LC3-II/LC3-I ↑;VEGF↓;PEDF↑	Promotion of mitophagy	NGR1 reduces Müller cells apoptosis, inhibits inflammatory cytokines release, increases retinal thickness, alleviates vascular leakage, and protects BRB integrity.	(123)
Alc	High-fat diet + STZ-induced SD rats (Alc, 16 mg/kg/day)	PINK1/Parkin↑; MDA↓;SOD, GPx↑;NLRP3↑	Promotion of mitophagy	Alc restores retinal layer thickness, alleviates vascular lesions, reduces retinal ganglion cell degeneration, protects BRB structural integrity, and decreases permeability.	(124)
Heyingwuzi formulation	HRCECs/STZ-induced C57BL/6 diabetic mice (treated with 10% HYWZF serum/administered with 12 g/kg or 24 g/kg HYWZF)	HIF-1α/BNIP3/NIX↑;LC3II/LC3I ↑;P62↓;claudin-5↑;VEGF↓	Promotion of mitophagy	HYWZF reduces RECs apoptosis, restores their function, enhances tight junctions, reduces BRB permeability, restores retinal thickness, and decreases acellular capillaries.	(126)

### 5.1 Inhibiting mitophagy in BRB constituent cells to improve BRB damage in DR

Melatonin, the primary hormone of the pineal gland, is also secreted by RECs (116). Functioning as an intracellular antioxidant and modulator of mitochondrial bioenergetic function, it traverses mitochondrial membranes, supporting its potential as a therapeutic agent for mitochondrial dysfunction-related diseases such as DR (117). In an *in vitro* study of a DME model, melatonin was found to reduce the expression of HIF-1α, HIF-1β, VEGF, and VEGF receptor genes, thereby preventing increased cell permeability and damage to the oBRB (118). Additionally, melatonin inhibits the expression of mitophagy-related genes (PINK, BNIP3, and NIX), thereby preventing excessive activation of mitophagy and maintaining mitochondrial homeostasis (119), reducing RPE cells apoptosis and alleviating BRB leakage. HG upregulates TXNIP in both rMC-1 cells and Müller cells of diabetic rats, leading to excessive activation of mitophagy. Knocking out TXNIP using CRISPR/Cas9 or intravitreal injection of TXNIP siRNA can inhibit this excessive activation. This intervention reduces Müller cell apoptosis and mitigates gliosis mediated by Glial Fibrillary Acidic Protein (GFAP) overexpression, potentially helping to maintain the supportive function of Müller cells for the BRB and mitigate structural damage to the BRB in diabetic retinopathy (98). HG also promotes apoptosis in ARPE-19 cells through upregulation of TXNIP. Knocking out TXNIP using TXNIP short hairpin RNA can significantly inhibit the excessive enhancement of mitophagy flux induced by high-glucose and alleviate mitochondrial fragmentation. This intervention restores the antioxidant function of thioredoxins (Trx1, Trx2), reduces ROS accumulation, alleviates increased lysosomal membrane permeability, and prevents the inactivation of tissue cathepsin L. Consequently, decreased RPE cells apoptosis and enhanced cell viability may contribute to maintained BRB structural integrity (66). Wnt inhibitory factor 1 (WIF1) is a gene reported to inhibit the Wnt/β-catenin signaling pathway. Initially discovered in human retinas, it is involved in regulating cell proliferation and tissue homeostasis. HG reduces the expression of WIF1, leading to excessive activation of mitophagy. Recombinant WIF1 protein downregulates the expression of mitophagy-related proteins in STZ-induced diabetic mice, including Parkin, PINK1, and the LC3-II/LC3-I ratio, inhibiting excessive activation of mitophagy (120). This helps restore RPE cells function, downregulate VEGFA expression, reduce tube formation in retinal endothelial cells, and maintain the integrity of the BRB.

### 5.2 Increasing mitophagy in BRB constituent cells to improve BRB damage in DR

In AGEs-induced Müller cells and STZ rat models, small extracellular vesicles from MSCs (which contain miR-125a-5p) activate PINK1/Parkin-mediated mitophagy by inhibiting PTP1B expression, significantly increasing the LC3-II/LC3-I ratio while decreasing levels of p62 protein and the tight junction protein occludin. Ultimately, these changes alleviate glial cell activation and reduce vascular leakage (101). Leflunomide acts on human retinal endothelial cells under high-glucose conditions *in vitro*, promoting the expression of the mitochondrial fusion protein mitofusin 2 (Mfn2), reversing Drp1-mediated excessive fission, restoring mitophagy flow, and increasing the expression of the tight junction protein ZO-1, which helps maintain the structural integrity of the BRB (65). Studies have shown that HG promotes Drp1 phosphorylation, leading to reduced mitochondrial fission, separation of HK-II from mitochondria, and inhibition of PINK1/Parkin-mediated mitophagy. Notably, Mdivi-1 (a Drp1 inhibitor) and rapamycin (an autophagy agonist) can reverse the above phenomena. Pretreatment with Mdivi-1 or rapamycin can reduce mitochondrial fission, enhance PINK1/Parkin-mediated mitophagy, decrease RMEC apoptosis and retinal vascular leakage, and reduce the number of acellular capillaries, thereby protecting BRB integrity (99). Research has shown that both in diabetic mice and under hyperglycemic conditions *in vitro*,TIN2 exacerbates the aging of RPE cells, TIN2 overexpression activates the mTOR signaling pathway and suppresses PINK1/Parkin-mediated mitophagy in ARPE-19 cells under high-glucose conditions. Conversely, knocking out TIN2 or using rapamycin reduced RPE cells aging, restored ZO-1 expression, increased retinal thickness, alleviated oxidative stress, and preserved BRB integrity (104).

Additionally, the decreased expression of SIRT3 caused by HG inhibits mitophagy. *In vitro* studies, overexpression of SIRT3 enhanced mitophagy in ARPE-19, reduced ROS production, decreased RPE cells apoptosis, and potentially maintained the barrier function (103). HG conditions *in vitro* induces the upregulation of Poldip2 expression in microglia, which directly obstructs the initiation and execution of mitophagy, leading to the accumulation of damaged mitochondria that cannot be cleared in time. Interventions targeting Poldip2 (such as knocking out Poldip2) can restore the activity of the AMPK/ULK1/PINK1 pathway, enhance mitophagy to clear damaged mitochondria, increase M2 polarization of microglia, reduce inflammatory cytokines, decrease retinal vascular leakage, and inhibit ocular neovascularization (lowering VEGFR) (100). Similarly, research teams have found that the membrane G protein-coupled bile acid receptor 5 (TGR5) enhances mitophagy and inhibits mitochondrial fission by regulating the PKCδ/Drp1-HK2 signaling pathway in STZ-induced SD rat models and HG-induced human retinal endothelial cells (RMEC). This mechanism reduces retinal vascular leakage, decreases the number of acellular capillaries, restores retinal thickness, reduces endothelial cell apoptosis, and maintains BRB integrity (121). Subsequently, in HG-induced human retinal capillary endothelial cells (HRCECs), increased expression of Drp1, decreased expression of MFN2, increased mtROS, and reduced expression of PINK1, Parkin, and VDAC1 proteins were observed. Further studies indicated that overexpression of VDAC1 could promote PINK1 expression and inhibit NLRP3 activation. Therefore, it is concluded that VDAC1 may be a potential target for the prevention and treatment of DR (122).

Ginsenoside R1 (NGR1) is a novel saponin extracted from Panax notoginseng with pharmacological properties. NGR1 pretreatment upregulates the levels of PINK1 and Parkin in db/db mouse retinas. It also increases the LC3-II/LC3-I ratio and downregulates the levels of p62/SQSTM1. These changes collectively enhance mitophagy via the PINK1/Parkin pathway (123). In these models,NGR1 reduces Müller cell apoptosis, lowers VEGF levels, increases PEDF expression, and inhibits the release of inflammatory cytokines. These preclinical findings demonstrate that NGR1 improves retinal function, increases retinal thickness, and attenuates vascular leakage under experimental conditions, thus potentially exerting protective effects on BRB integrity. Allicin (Alc) is a natural compound found in garlic that is gaining attention for its antioxidant and anti-inflammatory properties. Studies have shown that Alc promotes the expression of mitophagy-related proteins such as PINK1 and Parkin in DR rats, enhances mitophagy, reduces pro-inflammatory cytokines levels, and alleviates oxidative stress (124). These effects restore the thickness of various retinal layers, reduce vascular lesions, and decrease degeneration of retinal ganglion cells, thereby lowering BRB permeability. Recent studies have indicated that the HIF-1α/BNIP3/NIX pathway is associated with restoring autophagy in degenerated retinas, alleviating oxidative stress, and preventing diabetic retinopathy (125, 126). The traditional Chinese medicine Heyingwuzi formulation (HYWZF) inhibits excessive ROS production, cell apoptosis, tube formation, and invasion in HG-induced HRCECs by promoting mitophagy. After HYWZF treatment, the expression levels of the tight junction protein claudin-5, HIF-1α, Beclin1, BNIP3, and BNIP3L in mice were significantly higher than in the model group. The results indicate that HYWZF increases mitophagy through the HIF-1α/BNIP3/NIX axis, reduces apoptosis of retinal endothelial cells, increases tight junction protein levels, downregulates VEGF, decreases the number of acellular capillaries, reduces BRB permeability, and alleviates retinal tissue damage (126).

## 6 Discussion

Mitophagy is a selective degradation mechanism targeting dysfunctional mitochondria. It participates in mitochondrial quality control and maintains cellular homeostasis. Current evidence indicates that dysfunctional mitophagy is associated with multiple diseases, including DR. Therefore, targeting the mitophagy pathway may hold therapeutic potential.

We summarize the effects of targeting mitophagy on BRB in DR. The results show that under high-glucose conditions, mitophagy in cells exhibits bidirectional changes, rather than simply increasing or decreasing. Mitophagy may be excessively activated, leading to the clearance of necessary organelles and proteins, causing cells to lose compensatory capacity and ultimately undergo apoptosis. Conversely, a decrease in mitophagy can lead to the accumulation of damaged mitochondria. Whether through excessive activation or inhibition, either condition can ultimately lead to BRB degradation. This difference may be owing to variations in induction methods and the use of different animal or cell models across various studies, leading to inconsistent results. Mild hyperglycemia can induce a stress response that excessively activates mitophagy, leading to the clearance of damaged mitochondria. Conversely, severe or persistent hyperglycemia leads to cellular damage, resulting in mitophagy dysfunction. Additionally, the duration of diabetes affects mitophagy function. With prolonged diabetes duration, mitophagy gradually decreases, which may be related to aging, as aging inhibits its activation.

Mitophagy acts as a key regulator in DR. Existing drug and target interventions can improve BRB damage in DR by promoting or inhibiting mitophagy, providing an effective strategy for the precise regulation of DR. Current research has confirmed this feasibility through cellular and animal models (such as rats and mice), demonstrating high translational value. In particular, MSC-EVs therapy has reached the *in vitro* validation phase, with preliminary evidence demonstrating its ability to penetrate the BRB and deliver miR-125a-5p to Müller cells, potentially circumventing systemic drug side effects. Leflunomide, an FDA-approved anti-rheumatic drug, has been shown in animal studies to activate Mfn2 and ameliorate DR pathology, suggesting potential for accelerated clinical translation. Moreover, preclinical studies suggest that targeting molecules such as TXNIP, Drp1, and TIN2, or applying bioactive natural compounds like NGRI and Alc, may exert therapeutic potential by modulating mitophagic imbalance in BRB cells. These strategies merit further mechanistic and efficacy validation in subsequent research. Future research should focus on several aspects. First, retinal imaging technologies (such as OCT-A) should be combined to assess the state of mitophagy in patients and guide drug selection. Second, BRB-specific drug delivery systems should be developed, such as nanoparticles targeting Müller or RPE cells. Third, integrating multi-omics technologies to screen for new targets, such as compounds derived from the TXNIP pathway, can enhance treatment specificity and reduce side effects to support the development of DR treatment.
